# Efficacy of Geumguesingi-hwan (jinkuishenqi-wan, kinkijinki-gan) in decreasing blood glucose levels in patients with uncomplicated type 2 diabetes: A protocol for systematic review and meta-analysis

**DOI:** 10.1097/MD.0000000000029079

**Published:** 2022-03-25

**Authors:** Hyun-Jin Choi, Ji-Soo Baek, Cheon-Hoo Jeon, Seon-Mi Shin, Chung-Sik Cho

**Affiliations:** a *College of Korean Medicine, Daejeon University, Daejeon, Republic of Korea,*; b *Department of Internal Korean Medicine, College of Korean Medicine, Dae-Jeon University, Daejeon, Republic of Korea,*; c Department. of Internal Medicine, College of Korean Medicine, Se-Myung University, Jecheon, Republic of Korea.

**Keywords:** Geumguesingi-hwan, jinkuishenqi-wan, kinkijinki-gan, protocol, systematic review, type 2 diabetes

## Abstract

**Background:**

The purpose of a systematic review and meta-analysis is to verify the clinical efficacy and safety of Geumguesingihwan for patients with uncomplicated type 2 diabetes.

**Methods:**

The systematic review and meta-analysis will be performed following the guidelines of the National Evidence-based Healthcare Collaborating Agency. We will conduct a systematic search of randomized controlled trials in 8 electronic databases until August 31, 2021.

**Results:**

This study will provide evidence regarding the clinical efficacy of Geumguesingi-hwan from the following 3 perspectives: improving blood glucose level, insulin resistance, and β-cell function. Additionally, we will examine the safety of Geumguesingi-hwan by evaluating the adverse effects.

**Conclusions:**

This study will verify the antidiabetic efficacy and safety of Geumguesingi-hwan in patients with uncomplicated type 2 diabetes.

## 1. Introduction

Type 2 diabetes is a chronic metabolic disease characterized by increased insulin resistance and a gradual decrease in insulin secretion resulting from various causes. Chronic metabolic disorders are not only major mortality causes but can also cause serious complications.^[1]^

There has been an increase in the global impact of diabetes. Diabetes affected 463 million people worldwide in 2019, with this number being expected to reach 578 million by 2030 and 700 million by 2045.^[2]^ In 2019, the diabetes prevalence in Korea is 14.7% in men and 10.9% in women, which demonstrates the increasing diabetes prevalence in Korea.^[3]^ Accordingly, there has been an increase in the recognition rate and treatment rate of diabetes due to systematic screening tests and treatment guidelines. The diabetes awareness among individuals aged > 30 years increased by 3.2% from 68.3% in 2005 to 71.5% in 2016 to 2018, while the treatment rate increased by 17.2% from 49.0% to 66.2%.^[4]^

Unfortunately, there remains no optimal cure for diabetes; therefore, diabetes prevention is of emerging importance. Patients newly diagnosed with diabetes should start systematic diet and exercise plans as well as regularly monitor and manage their blood sugar. In case of severe hyperglycemia (glycated hemoglobin > 9.0%) accompanied by hyperglycemia symptoms, including polyuria and weight loss, hypoglycemic agents or even insulin injection are used to lower blood sugar.^[1]^

There have been studies on treatment methods in East Asian Traditional Medicine (EATM) for lowering blood sugar levels and its complications in patients with diabetes. Son et al^[5]^ confirmed the antidiabetic effect of various herbal medicines, which suggested the possibility of expanding the field of EATM. Additionally, Jeong reported that fasting and postprandial blood glucose levels were improved by the administration of Jowiseunggi-tang, Dashiho-tang, Bojungikgi-tang, and Hoechunyanggyeok-san to patients with type 2 diabetes presenting with thirst and frequent urination.^[6]^

Geumguesingi-hwan (GSH), in which Rehmannia radix is a principal medicine, is a prescription used to improve various symptoms caused by Yang deficiency syndrome in the kidney.^[7]^ A meta-analysis published by Chen provided clinical evidence regarding the antidiabetic effects of GSH in patients with type 2 diabetes mellitus showing diabetic neuropathy.^[8]^ Another systematic review conducted in 2015 demonstrated the treatment efficacy of GSH in patients with diabetic complications. However, there have been no further studies on the therapeutic effect of GSH in patients with uncomplicated type 2 diabetes.^[9,10]^

Therefore, we aim to conduct a systematic review of the existing literature regarding the hypoglycemic effect of GSH in patients with uncomplicated type 2 diabetes as well as to verify the clinical efficacy and safety of GSH.

## 2. Methods

### 2.1. Protocol registration

This protocol is registered in the Open Science Framework (OSF), with the registration doi 10.17605/OSF.IO/Z93CW (https://osf.io/z93cw), and will be reported following the Preferred Reporting Items for Systematic Reviews and Meta-analyses guidelines.

### 2.2. Eligible criteria for study selection

#### 2.2.1. Types of studies.

We will only include systematic reviews of randomized controlled trials and exclude case reports, case series, meta-analyses, and animal testing.

#### 2.2.2. Types of participants.

We will select patients with clinical symptoms, including polyuria, polygamy, and weight loss, or those diagnosed with type 2 diabetes through blood sugar tests. We will exclude patients with diabetes-related complications, including neuropathy, obesity, and retinopathy, as well as those with gestational diabetes and type 1 diabetes. There will be no restrictions regarding age and sex.

### 2.3. Types of interventions

#### 2.3.1. Experimental interventions.

We will include various formulas of GSH, including decoctions, capsules, granules, and powder, as well as modified prescriptions in case the author has mentioned that it originated from GSH.

#### 2.3.2. Control interventions.

We will include studies that used GSH with conventional treatment as the experimental intervention or compared GSH with conventional treatment as a control group. The control interventions of the included studies will comprise placebo, conventional treatment, waiting-list control, or no treatment.

### 2.4. Types of outcomes

We will include the following main outcomes: fasting glucose level, 2-hour postprandial glucose level, hemoglobin A_1C_, fasting insulin, homeostatic model assessment for insulin resistance, and homeostatic model assessment of β-cell function. Additional outcomes will include the clinical effective rate and adverse events.

#### 2.4.1. Searching strategy.

For Pubmed, Cochrane Library, and EMBASE, we will use the following search terms: “Diabetes Mellitus, Diabetes, T2DM, NIDDM, non-insulin dependent diabetes mellitus” and “Geumguesingihwan, jinkuishenqi-wan, kinkijinki-gan”. The search will proceed as follows: {(Diabetes Mellitus OR Diabetes OR T2DM OR NIDDM OR non-insulin dependent diabetes mellitus) AND (Geumguesingihwan OR jinkuishenqi-wan OR kinkijinki-gan)}. In the CNKI and CiNii, we will use the following combination of keywords: “jinkuishenqi-wan, kinkijinki-gan” and “Type 2 diabetes”. Finally, in the domestic database, we will combine “Type 2 diabetes” and “Geumguesinkihwan”, followed by exclusion of studies by reviewing the title and abstract (Table 1).

**Table 1:** Search strategy for the PubMed.

**No.**	**Search terms**
#1	“Diabetes Mellitus”[MeSH Terms]
#2	“Diabetes *”[Title/Abstract]
#3	“Diabetes”[Title/Abstract] OR “T2DM*”[Title/Abstract] OR “NIDDM”[Title/Abstract] OR “Type 2”[Title/Abstract] OR “Type II”[Title/Abstract]
#4	“non insulin* depend*” [Title/Abstract] OR “non-insulin* depend*”[Title/Abstract] OR “non insulin?depend*”[Title/Abstract] OR “non-insulin?depend*”[Title/Abstract]
#5	#1 OR #2 OR #3 OR #4
#6	“Geumguesingi-hwan”[Title/Abstract]
#7	“Jinkuishenqi-wan”[Title/Abstract]
#8	“kinkijinki-gan”[Title/Abstract]
#9	#6 OR #7 OR #8
#10	#5 AND #9

### 2.5. Data collection

#### 2.5.1. Study selection.

**Figure 1.**
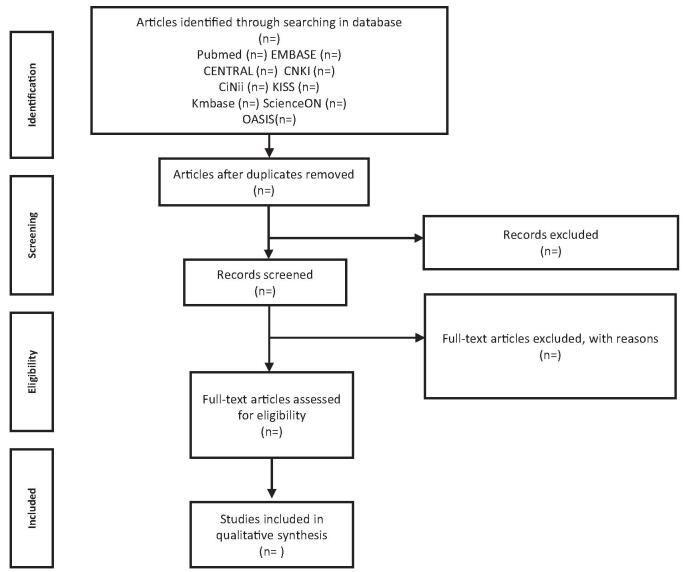
PRISMA flow of selection process.

We will import all collected articles, using the reference management software Endnote 20.0.1 software (Clarivate Analytics, Boston, MA). Next, we will remove all duplicate articles. And then, 2 independent researcher (HJC, JSB) will screen the studies based on the inclusion and exclusion criteria. Initially, papers will be screened by reviewing the title and abstract; further, papers with missing original text will be excluded. Subsequently, selected papers will undergo a full-text review by both researchers. Disagreements between the reviewers will be resolved through discussions with a third researcher (CHJ) (Fig. 1).

#### 2.5.2. Data extraction.

We will extract the following data from the studies: research information (first author, publication year, and publication region), research participants (total number of participants, ratio of men and women, etc), interventions (intervention method and process, number of participants, etc), comparative interventions (comparative intervention methods and processes, number of participants, etc), outcome (evaluation index, result value, etc), and side effects. The extracted information will be summarized and organized using Microsoft Excel 2020.

### 2.6. Data analysis

#### 2.6.1. Risk of bias assessment.

Bias is a systematic error that indicates the deviation of the result or estimation from the true value, which may lead to underestimation or overestimation of the intervention effect. We will assess the risk of bias assessment using Cochrane's Risk of Bias (RoB) tool following the guidelines of the National Evidence-based Healthcare Collaborating Agency. The RoB tool evaluates bias in the following 7 areas: random sequence generation, allocation concealment, blinding of participants and researchers, blinding of outcome assessment, insufficient outcome data, selective reporting, and other potential risk of bias. To reduce differences in opinion resulting from interpretation, a tool translated into Korean will be used. Based on the study content, the probability of bias in each area will be evaluated as high or low risk of bias. If it is difficult to determine the bias possibility, we will evaluate it as unclear risk of bias. Two independent researchers (HJC, JSB) will conduct the evaluation, with disagreements being resolved through discussions with a third researcher (CHJ).

#### 2.6.2. Meta-analysis.

A meta-analysis will be performed to yield meaningful conclusions regarding the clinical effects of GSH. The analysis will be conducted using RevMan (Review Manager) 5.4 version of (Copenhagen, The Nordic Cochrane Center, the Cochrane Collaboration). Continuous data will be presented as the mean and standard deviation (SD). For dichotomous data, we will perform a meta-analysis using the risk ratio (RR) with a 95% confidence interval (CI).

#### 2.6.3. Missing data.

In case of missing data, the author will be contacted for pertinent information. In case the complete data are inaccessible, this will be recorded in the risk of bias assessment; moreover, data analysis will be conducted only using the available data.

#### 2.6.4. Heterogeneity assessment.

In meta-analysis, heterogeneity refers to when the variation in the results of each study is more than the sampling error and cannot be explained by chance. Higgin's *I*^2^ statistic, which quantifies the inconsistency, will be used to evaluate statistical heterogeneity. An *I*^2^ value of 0% to 40%, 30% to 60%, 50% to 90%, and 75% to 100% indicated non-significant, moderate, substantial, and significant heterogeneity, respectively.

#### 2.6.5. Assessment of reporting biases.

To visually evaluate the reporting bias, we will use funnel plots for meta-analysis including > 10 trials.

#### 2.6.6. Data synthesis.

Fixed-effect and random-effects models will be used in case of homogeneity and heterogeneity, respectively.

### 2.7. Ethics and dissemination

This protocol did not require ethical approval since this systematic review did not use individual patient data. The results will be published in a peer-reviewed journal and presented at relevant conferences.

## 3. Discussion

Given the rapid increase in the worldwide prevalence of diabetes, there is an increase in the treatment cost burden due to the low treatment rate and control rate.^[11]^ There remains no effective diabetes treatment; therefore, there have been continuous efforts to develop novel treatment strategies for diabetes.

GSH, which is also termed as baweidihuang-wan, is a prescription first mentioned in the ancient literature “jinguiyaolue”. Its basic indications include symptoms caused by Yang deficiency in the kidney system, such as weakness in the lower extremities, chills, frequent urination, back pain, diarrhea, cold in the extremities, tinnitus, lethargy, edema, renal colic, erectile dysfunction, and decreased sexual desire.^[7]^ A previous study found that GSH decreased serum leptin levels and insulin resistance index as well as increased insulin sensitivity in rats. Additionally, GSH can significantly decrease the levels of fasting blood glucose, glucagon, total cholesterol, triglyceride, and lowdensity lipoprotein in rats.^[12,13]^ Moreover, GSH can significantly reduce 24-hour urine protein, blood urea nitrogen, and serum creatinine levels in the treatment of diabetic nephropathy.^[8]^

Since 2015, there has been no meta-analysis on diabetes alone, with Chen et al^[8]^ conducting the most recent metaanalysis of the use of GSH for diabetic nephropathy.^[9]^ Therefore, we will perform a systematic review and meta-analysis of the hypoglycemic effect of GSH in patients with uncomplicated type 2 diabetes.

This study has several potential limitations. First, standardized dosing is difficult due to differences in the dosage, frequency, and administration method; additionally, there may be differences in the degree of blinding when using different formulations. Additionally, there could be publication bias, information bias, and poor statistics. To overcome this limitation, 2 independent reviewers will conduct a systematic meta-analysis, with disagreements being resolved through a third researcher.

## Acknowledgments

The authors would thank Editage (www.editage.co.kr) for English language editing.

## Author contributions

**Conceptualization:** Chung-Sik Cho and Seon-Mi Shin.

**Data curation:** Hyun-Jin Choi and Ji-Soo Baek.

**Formal analysis:** Hyun-Jin Choi, Ji-Soo Baek, and Chung-Sik Cho.

**Funding acquisition:** Chung-Sik Cho and Seon-Mi Shin.

**Writing** - **original draft:** Hyun-Jin Choi and Ji-Soo Baek.

**Writing** - **review & editing:** Hyun-Jin Choi, Cheon-Hoo Jeon, Seon-Mi Shin, and Chung-Sik Cho.
